# Caste-Based Inequality in Fruit and Vegetable Consumption in India

**DOI:** 10.1177/03795721211026807

**Published:** 2021-07-19

**Authors:** Samira Choudhury, Bhavani Shankar, Lukasz Aleksandrowicz, Mehroosh Tak, Alan Dangour

**Affiliations:** 1 College of Medicine and Health, University of Exeter, Exeter, United Kingdom; 2 Institute for Sustainable Food, The University of Sheffield, Sheffield, United Kingdom; 3 228093 Department of Population Health, London School of Hygiene & Tropical Medicine, London, United Kingdom; 4 Leverhulme Centre for Integrative Research on Agriculture and Health (LCIRAH), London United Kingdom

**Keywords:** India, fruit and vegetable consumption, nutrition, caste-based inequality, regression decomposition

## Abstract

**Objective:**

Fruit and vegetable (F&V) consumption is of central importance to many diet-related health outcomes. In India, caste is a major basis of socioeconomic inequality. Recent analysis shows that more disadvantaged “lower” castes consume less F&V than the rest. This article explores whether this consumption gap arises due to differential distribution of drivers of consumption such as income and education across castes, or whether behavioral differences or discrimination may be at play.

**Design:**

The Oaxaca-Blinder regression decomposition is applied to explain the gap in F&V consumption between “upper” castes and “lower” castes, using data from the 68th (2011-2012) round of the National Sample Survey Organization household survey.

**Results:**

Differences in the distribution of F&V drivers account for all of the 50 grams/person/day consumption gap between upper and lower castes. In particular, much of the gap is explained by income differential across castes.

**Conclusions:**

In the long run, India’s positive discrimination policies in education and employment that seek to equalize income across castes are also likely to help close the F&V consumption gap, leading to health benefits. In the medium run, interventions acting to boost lower caste income, such as cash transfers targeting lower castes, may be effective.

## Introduction

Caste represents a major basis of socioeconomic inequality in India. The official classification refers to 4 major categories of caste: Scheduled Tribes (STs), Scheduled Castes (SCs), Other Backward Castes, and Other (“Forward”) Castes (the use of the terminology “lower castes” to refer to SCs and Tribes, and “upper castes” to refer to “other” and “other backwards” castes is also common in India). Communities belonging to “lower castes,” that is, STs and SCs, are the most disadvantaged social groups in India.^1^ Approximately 25% of the Indian population is classified as belonging to a SC or a ST.^2^ Recognizing their relative social and economic deprivation, the Indian constitution has historically accorded special status to SCs and STs and put in place positive discrimination measures in education and employment.^3^ Despite such measures and an improvement in their position over time, SC/STs continue to suffer multiple disadvantages relative to the remaining population.^4-7^

The disadvantage suffered by SC/STs in India is known to extend to several dimensions relating to health and nutrition as well. Children from lower castes have been shown to have higher rates of infant mortality and lower iron and vitamin supplementation rates, in addition to displaying relatively lower use of public health services, compared to children from upper castes.^7^ Scheduled Caste/Scheduled Tribe children have been found to have lower height-for-age on average compared to children from upper castes, and this has been linked to lower use of health services and worse parental education.^6^ The literature has also explored gaps between SC/STs and upper castes in the use of maternal health care, highlighting disparities in antenatal coverage.^8^

The above literature suggests that caste represents a major basis for inequality across a wide range of health and nutrition indicators in India. However, much less is known about whether caste also impinges upon key dietary and food consumption outcomes of importance to health. The Global Burden of Disease project^9^ ranks dietary risks second among major risk factors for death and disability in India. Achieving adequate fruit and vegetable (F&V) consumption is a fundamental priority in India, where both micronutrient deficiencies and diet-related noncommunicable diseases have high prevalence.^10,11^ However, recent analyses have shown that average F&V consumption in the Indian population is worryingly low^12^ and that lower castes appear to consume significantly less F&V than upper castes.^13^

In this article, we attempt to unpack the relationship between caste and F&V consumption. Lower castes may display lower F&V consumption as a result of being endowed with lower levels of important drivers that boost F&V consumption, such as income or education (a “covariate” effect in a regression context). Or their relatively lower consumption may be a result of a weaker *strength of relationship* between drivers and F&V consumption outcomes (a “coefficient” effect). Sources of such coefficient effects may include behavioral aspects (eg., increase in income translates into a lower boost to F&V consumption for some groups because of cultural/behavioral aspects) or some form of “discrimination” (eg., some groups may live in areas worse served by food markets, resulting in a weaker relationship between drivers and F&V consumption outcomes). Previous research has suggested that food consumption and its evolution in India is subject to strong sociocultural influences,^14,15^ and that caste plays a role in dietary patterns.^16^ Is the lower F&V consumption of SC/STs in India a result of covariate effects or coefficient effects, or both? Such a question has important implications for policy and strategy. If this consumption gap arises because of endowment disparities, then policies, traditional development strategies that aim to equalize incomes across castes, such as caste-based education and employment policies also work toward equity in F&V consumption. However, if intercaste differences in the strength of relationship between drivers and F&V consumption play a key role, more targeted policies may need to be designed, such as behavior change communication strategy aimed at improving F&V consumption, particularly among lower castes. This article aims to shed light on this research question using the Oaxaca-Blinder (OB) regression decomposition method.

## Data

We use data from the latest available 68th (2011-12) round of India’s National Sample Survey Organization’s (NSSO) household survey. The NSSO is a nationally representative multipurpose repeated cross-sectional survey that collects information on household expenditure and consumption (note 1).

Our analysis uses food consumption data collected using a 7-day recall method by NSSO 2011 to 12. The NSS asked household respondents to recall the total consumption of F&V by the household during the reference period, including consumption from home production (note 2) households, respectively. After excluding households with outlier values of per capita calorie intake, our final estimation sample contains 98 879 households, with 70 272 households belonging to the upper castes (“other” and “backward” castes) (71%) and 28,607 households belonging to SC/STs (29%). Our dependent variable is household F&V consumption (g/adult equivalent/day), which is the sum of fruits and vegetables (excluding tubers and pulses) consumed at home by the household in the last 7 days, divided by the sum of household adult equivalent units. Each household member was assigned an adult equivalent unit, which is the ratio of that member’s age- and sex-specific recommended daily dietary energy, compared to an adult male. For each household’s consumption, we also include (1) meals prepared at home but consumed by nonmembers and (2) meals received for free from other households by household members. The NSS also records limited information on out-of-home consumption of meals/snacks. However, this is based on a single respondent’s estimation on behalf of the entire family and is of questionable reliability.^17-19^ Therefore, we do not include out-of-home consumption in our calculations (note 3).

Our set of covariates includes household-level economic and sociodemographic indicators that have been linked to household dietary outcomes in previous literature.^13,20,21^ In particular, we follow the specification recently used by Choudhury et al.^13^ To proxy income, we use per capita monthly expenditure (note 4). To estimate price (“unit values”), we divide reported expenditure by purchased quantity, and we do this for all foods combined, as well as separately for F&V (note 5). We then compute the relative price of F&V as the ratio of F&V unit value over the unit value for all foods. Level of education, measured as years of schooling of the female spouse, is used to proxy nutritional knowledge.^22,23^ We also include a dummy variable representing female-headed households, based on the research^24^ which suggests that the nutrition sensitivity of household resource allocation is gender dependent.

Given the prominence of sociocultural patterns in Indian diets,^14^ we use a binary variable to specify whether households are Hindu or not. To further account for regional and cultural heterogeneity, we include a set of state-level dummy variables. Food consumption can derive from 2 broad sources, household production (and local exchange), or market purchase. A systematic review of this literature finds a positive association between market access and household food consumption.^25^ However, the literature also argues that agricultural production and proximity to agricultural production are linked with household food consumption outcomes in many low-income settings since markets are often weak, posing challenges to market sourcing of foods.^26^ Therefore, we include covariates to indicate whether a household is rural or urban and whether it is primarily employed in the agriculture sector.

## Methods

We use regression decomposition methods in the form of the OB decomposition of the differences in mean F&V consumption between SC/STs and the upper castes (“other” and “backward” castes). The OB decomposition has the advantage of examining the gap in mean outcomes between the 2 population groups.^27,28^ It not only quantifies how much differences in the levels of key drivers explain the F&V consumption gap between upper and lower castes but also identifies how intercaste differences in relationships between endowments and F&V consumption explain the gap. The OB decomposition partitions the mean gap in F&V consumption between the upper castes and SC/STs into 3 parts: a part that is due to the differences in the levels of key drivers between the 2 groups (covariate effects); a second part that is due to the differences between the groups in the strength of relationships between drivers and F&V consumption (coefficient effects); and a third part that arises from interactions between covariate and coefficient effects.

To show this more formally, suppose F&V consumption is explained by only one driver *x*, based on a linear regression model. We have 2 groups labeled “*lc*” for “lower castes” (SC/ST) and “*uc*” for “upper castes” (other/backward castes), respectively. The relationship between *x* and F&V consumption is allowed to vary between the upper castes and lower castes:


$$F\&V^{uc}={\text{ β}}^{uc}x^{uc}+{\text{ε}}^{uc}\text{ if household belongs to }upper\;castes\;\left(other/backward\right)$$

$$F\&V^{lc}={\text{ β}}^{lc}x^{lc}+{\text{ε}}^{lc}\text{ if household belongs to }lower\;castes\;\left(SC/ST\right)$$

Then, the gap in F&V consumption between the upper castes and lower castes can be expressed as:


$${\bar{F\&V}}^{uc}-\;{\bar{F\&V}}^{lc}={\text{ β}}^{uc}{\bar{x}}^{uc}-{\text{ β}}^{lc}{\bar{x}}^{lc}$$

Where ${\bar{F\&V}}^{uc}$ and ${\bar{F\&V}}^{lc}$ represent average F&V consumption in each group, ${\bar{x}}^{uc}$ and ${\bar{x}}^{lc}$ are the average endowments of driver *x* for each group, and ${\text{β}}^{uc}$ and ${\text{β}}^{lc}$ are the relationships between *x* and F&V consumption for the 2 groups. Therefore, the gap in F&V consumption between the 2 groups can be written as


$$\begin{array}{l} {\bar{F\&V}}^{uc}-{\bar{F\&V}}^{lc}=\left({\bar{x}}^{uc}-{\bar{x}}^{lc}\right){\text{β}}^{lc}+\left({\text{β}}^{uc}-{\text{β}}^{lc}\right){\bar{x}}^{lc}+\left({\bar{x}}^{uc}-{\bar{x}}^{lc}\right)\left({\text{β}}^{uc}-{\text{ β}}^{lc}\right)\\ =\text{Δ}x{\text{β}}^{lc}+\text{Δβ}x^{uc}+\text{Δ}x\text{Δβ} \end{array}$$

The above equation says that the gap in F&V consumption between lower castes and upper castes is comprised of 3 components. The first part, $\text{Δ}x{\text{β}}^{lc}$ is the difference in consumption arising from differential endowments of the driver *x* across the 2 groups (the covariate effect). The second part, $\text{Δβ}x^{uc}$ is the consumption differential arising from differences in coefficients between *uc* and *lc* (the coefficient effect). The third term ΔxΔβ is the interaction between the covariate and coefficient effects. It can be shown that the decomposition also generalizes to the multivariate regression case.

We estimated the regressions and computed the OB decompositions as indicated above, using the data and regression specification described in the previous section.

## Results

### Descriptive Statistics

Table 1 reports sample means for all variables in the regressions by caste groupings. Significant differences between upper and lower castes based upon *t* tests of group means are also indicated. Table 1 reveals that there is a 50 g/adult equivalent/day gap in average F&V consumption between the lower castes (253 g) and upper castes (303 g). Indeed, the F&V consumption among both groups is below the Indian National Institute of Nutrition^29^ and the WHO’s Global Strategy on Diet, Physical Activity and Health recommendation of 400 g of F&V/day. Table 1 also suggests substantial differences in some of the key drivers of F&V consumption between caste groups; particularly that upper caste households have much higher income (expenditure), are more educated, and less likely to live in rural areas, compared to lower castes.

**Table 1.** Sample Means of Household Fruit and Vegetable Consumption and Its Drivers Across Total Population, Upper Castes, and Lower Castes Variable.^a^

	Total population	Upper castes	Lower castes
F&V consumption (g/adult equivalent/day)	284.05	302.65^b^	252.88
Per capita monthly consumer expenditure (Rs)	2006.39	2207.71^b^	1488.97
Relative price of F&V(ratio of F&V unit value (price) relative to all foods)	1.94	1.94^b^	1.93
Household size	4.45	4.44^b^	4.47
Number of children younger than 5	0.46	0.44^b^	0.49
Years of education of female spouse	3.46	3.79^b^	2.61
Female headed households (%)	11.45	11.47	11.38
Rural location (%)	68.94	64.52^b^	80.30
Agricultural households (%)	49.69	49.15^b^	51.08
Hindu (%)	83.05	80.18^b^	90.46
Observations	98 879	70 272	28 607

Abbreviation: F&V, fruit and vegetable.

^a^ Results of *t* tests of differences between upper and lower castes are indicated in asterisks. Source: computed using NSS (2011-2012).

^b^ *P* < .01.

^c^ *P* < .05.

^d^ *P* < .1.

### Oaxaca-Blinder Decomposition Results

Table 2 presents results from the ordinary least squares regression models estimated separately for lower castes, upper castes, and total population. Allowing all coefficients to vary by caste requires separate regressions by caste and thus, following the standard methodology, we first estimate separate regressions. These regressions are precursors to the OB decomposition computation.

**Table 2.** OLS Regression Estimates for Household Fruit and Vegetable Consumption (g/adult equivalent/day) by Total Population, Upper Castes, and Lower Castes.^a^

	Total population	Upper castes	Lower castes
Log per capita monthly consumer expenditure	159.22 (3.09)^b^	160.80 (1.32)^b^	153.18 (1.97)^b^
Log relative price of F&V	−13.72 (3.39)^b^	−18.08 (1.98)^b^	−3.05 (2.48)
Household size	−18.09 (0.51)^b^	−18.16 (0.34)^b^	−17.85 (0.46)^b^
Number of children younger than 5	17.13 (0.97)^b^	16.97 (0.88)^b^	17.45 (1.10)^b^
Years of education of female spouse	0.41 (0.34)	0.74 (0.20)^b^	−0.78 (0.30)^c^
Female headed households	68.00 (4.42)^b^	71.73 (2.14)^b^	58.42 (2.74)^b^
Rural location	26.32 (2.65)^b^	26.60 (1.47)^b^	25.24 (2.30)^b^
Agricultural households	9.47 (1.81)^b^	10.58 (1.25)^b^	6.13 (1.68)^b^
Hindu	1.43 (2.85)	1.03 (1.60)	−1.15 (3.24)
Caste (baseline: upper castes)			
SC/STs	−3.63 (2.14)^d^		
Observations	98 879	70 272	28 607
*R* ^2^	0.34	0.34	0.33

Abbreviations: F&V, fruit and vegetable; SC, Scheduled Caste; ST, Scheduled Tribe.

^a^ Source: estimated based data from NSS (2011-2012). Standard errors were adjusted for clustering given the multistage sampling design of the NSS survey.

^b^ *P* < .01.

^c^ *P* < .05.

^d^ *P* < .1.

The results suggest that income exerts a strong positive influence on F&V consumption. Fruit and vegetable consumption is also positively associated with lower F&V price relative to other foods, and with smaller household sizes. Rural location, agricultural occupation, and female household headship also have a positive relationship with F&V consumption. A difference in coefficient size across caste groups is evident for the income, price, and education variables. We also estimated a version of the whole sample regression where each covariate was interacted with a dummy variable for lower or upper caste (note 6).

Table 3 contains the results of the OB decomposition, showing the contribution of differences in the levels of drivers (covariate effects) and differences in the strength of relationships between drivers and F&V consumption (coefficient effects), respectively, in explaining the gap in F&V consumption between upper and lower castes. In aggregate, covariate effects are very dominant, accounting for almost 47 of the 50 grams gap between upper and lower castes in mean F&V consumption. Furthermore, Table 3 shows that the higher mean income of the upper castes compared to lower castes is overwhelmingly the major source of the F&V consumption gap, accounting for almost all of it. Although the relative endowments of lower castes in terms of their rural location, family size, and agricultural occupation act to lower the gap, these effects are very small compared to the income effect. In contrast to the covariate effects, in aggregate, coefficient differences explain very little of the mean gap in F&V consumption across upper and lower castes. The higher price sensitivity of upper caste F&V consumption compared to lower caste acts to narrow the F&V consumption gap. Other coefficient effects are small and/or statistically insignificant, as are the interaction effects.

**Table 3.** Oaxaca-Blinder Decomposition of Mean F&V Consumption Gap Between Upper Castes and Lower Castes.^a^

	Covariate effect		Coefficient effect		Interaction effect	
	Estimate	Standard error	Estimate	Standard error	Estimate	Standard error
Aggregate effect	46.77^b^	1.99	3.09	2.08	3.82^c^	1.78
Log per capita monthly consumer expenditure	49.92^b^	1.82	54.39	41.09	2.37	1.79
Log relative price of F&V	−0.08	0.10	−15.31^c^	6.98	−0.07	0.09
Household size	0.33	0.55	−1.41	4.82	0.01	0.02
Number of children under 5	−0.84^b^	0.20	−0.24	1.03	0.02	0.10
Years of education of female spouse	0.90^d^	0.49	4.06^c^	1.78	1.84^c^	0.81
Female headed households	0.24	0.31	1.46	0.97	0.04	0.06
Rural location	−4.14^b^	0.50	1.11	3.84	−0.21	0.74
Agricultural households	−0.20^c^	0.09	2.31	1.99	−0.09	0.08
Hindu	−0.11	0.34	1.97	6.42	−0.22	0.73

Abbreviation: F&V, fruit and vegetable.

^a^ Source: estimated based data from NSS (2011-2012). Standard errors were adjusted for clustering given the multistage sampling design of the NSS survey.

^b^ *P* < .01.

^c^ *P* < .05.

^d^ *P* < .1.

## Discussion and Conclusion

Despite decades of affirmative policy action, caste continues to have a persistent negative bearing on many human welfare outcomes in India, in particular, in the realm of health and nutrition. Children belonging to SCs/STs have been shown to suffer numerous disadvantages relating to health and nutrition outcomes in comparison with “upper” castes. Yet there has been surprisingly little attention devoted to potential caste-related disparities in Indian diets, which are a major proximate determinant of many nutrition and health outcomes. Choudhury et al. (2019) have recently raised the prospect of caste being a key source of inequality in F&V consumption in India, which holds implications for many diet-related health outcomes.

However, the question arises as to whether any observed caste-based differentials in F&V consumption arise primarily because lower castes have worse endowments of income, education, and other key drivers of F&V consumption, or whether they arise from differentials across castes in how F&V consumption responds to these drivers. Such a question has a potentially important bearing on policy and strategy: if endowment disparities are key, the focus can be on broad-based equalization of endowments such as income and education. In other words, traditional development strategies predominantly aimed at alleviating poverty among SC/STs would suffice to also equalize F&V consumption outcomes in the long run. But in contrast, if intercaste differences in relationships between endowments and F&V consumption are key, more targeted policies may be needed, for example, behavioral change communication aimed at encouraging F&V consumption in a particular caste or improving F&V availability in tribal areas. The OB regression decomposition that we apply is designed to shed light on the relative importance of these differences in the levels of drivers (covariate effects) and differences in the strength of relationships between drivers and F&V consumption (coefficient effects).

Our main findings are striking. Not only is much of the 50-gram F&V consumption gap between upper and lower castes explained by covariate effects, differences in incomes across caste groups alone largely account for the differential. In terms of policy implications for the long run, this finding supports the notion that historical and ongoing affirmative action policies in India may eventually have an equalizing effect on F&V consumption. As these policies primarily revolve around quotas for disadvantaged castes in educational institutions and public sector employment, they provide impetus toward equalizing incomes across castes and can thereby help close the F&V consumption gap. Another implication of our results is that, in the medium run, income support interventions that target SC/STs may help close the F&V consumption gap by promoting intercaste income equality. An example of this is the Mahatma Gandhi National Rural Employment Guarantee Scheme established in 2006, which aims to enhance the livelihoods of disadvantaged social groups, especially SCs/STs through guaranteed employment.^30,31^ In addition, the pilot unconditional cash transfer scheme that has been trialed by Sewa-Unicef has included tribal villages in its target population.^32,33^

The greater tendency of lower castes to be rurally based and engaged in agricultural production has a minor equalizing effect on the F&V gap between castes. Urban as well as rural F&V markets in India are generally weak given infrastructure limitations and the lack of cold chain facilities. However, rural location and proximity to agricultural production provide an alternative source of F&V access that may be less available to urban households. However, this effect is minor. Among the coefficient effects, the greater sensitivity of upper castes to F&V relative price changes appears to act to equalize F&V consumption. However, this as well as other coefficient effects have only minor or negligible implications for the consumption gap, suggesting that behavioral aspects may not be important in this setting. Income differences are the critical element.

The limitations of this study are fully acknowledged. The household, rather than individual, nature of the data is one limiting aspect. The multipurpose household survey nature of the NSSO data also constrains the covariate set that can be included in the analysis. The cross-sectional nature of the study implies that causality cannot be inferred from the estimated regressions. It is also important to emphasize that our OB decomposition is only able to explain mean gaps in F&V consumption. Further analysis may fruitfully explore decompositions based on quantile regression methods,^34^ which would enable insight into whether, for example, covariate or coefficient effects dominate at the lower tail of F&V consumption.

## Notes

1. The 2011-12 round followed a stratified design with districts from each state or union territory comprising the first stratum, subdivided into rural and urban sectors. Villages within these substrata comprised the first stage units. In the second stage, household samples were drawn from each first stage unit.

2. The original NSS sample included 101,651 (59,683 rural and 41,968 urban.

3. This may be a potential source of bias. Lower castes may have work further away from home since they are often in work involving manual labor, and therefore, it is possible that they consume more foods away from home.

4. Per capita monthly expenditure is based on the sum of total food consumption and total nonfood expenses.

5. The value of food items (Rs) and quantity of consumption (kg) from purchased and own production are asked separately and we have taken the sum of these as the total household intake and expenditure.

6. A joint F test of these interaction effects was computed, (F(42, 12 730) = 1.85) and found to be statistically significant at the 1% level, suggesting that coefficients as a whole do tend to vary by caste.
